# Prevention of sickness absence through early identification and rehabilitation of at-risk patients with musculoskeletal pain (PREVSAM): a randomised controlled trial protocol

**DOI:** 10.1186/s12891-020-03790-5

**Published:** 2020-11-28

**Authors:** MEH Larsson, L. Nordeman, K. Holmgren, A. Grimby-Ekman, G. Hensing, C. Björkelund, S. Bergman, A. Ekhammar, M. Dottori, S. Bernhardsson

**Affiliations:** 1 Department of Education, Research and Development Primary Health Care, Region Västra Götaland, Gothenburg, Sweden; 2 Unit of Physiotherapy, Department of Health and Rehabilitation, Institute of Neuroscience and Physiology, The Sahlgrenska Academy at University of Gothenburg, Gothenburg, Sweden; 3 Department of Education, Research and Development Primary Health Care, Region Västra Götaland, Borås, Sweden; 4 Unit of Occupational Therapy, Department of Health and Rehabilitation, Institute of Neuroscience and Physiology, The Sahlgrenska Academy at University of Gothenburg, Gothenburg, Sweden; 5 School of Public Health and Community Medicine, Institute of Medicine, The Sahlgrenska Academy at University of Gothenburg, Gothenburg, Sweden; 6 Primary Care, School of Public Health and Community Medicine, Institute of Medicine, The Sahlgrenska Academy at University of Gothenburg, Gothenburg, Sweden

**Keywords:** Musculoskeletal, Prevention, Rehabilitation, Interdisciplinary, Sick leave, Intervention, Randomised

## Abstract

**Background:**

Musculoskeletal pain is globally a leading cause of physical disability. Many musculoskeletal-related pain conditions, such as low back pain, often resolve spontaneously. In some individuals, pain may recur or persist, leading to ong-term physical disability, reduced work capacity, and sickness absence. Early identification of individuals in which this may occur, is essential for preventing or reducing the risk of developing persistent musculoskeletal pain and long-term sickness absence. The aim of the trial described in this protocol is to evaluate effects of an early intervention, the PREVSAM model, on the prevention of sickness absence and development of persistent pain in at-risk patients with musculoskeletal pain.

**Methods:**

Eligible participants are adults who seek health care for musculoskeletal pain and who are at risk of developing persistent pain, physical disability, and sickness absence. Participants may be recruited from primary care rehabilitation centres or primary care healthcare centres in Region Västra Götaland. Participants will be randomised to treatment according to the PREVSAM model (intervention group) or treatment as usual (control group). The PREVSAM model comprises an interdisciplinary, person-centred rehabilitation programme, including coordinated measures within primary health care, and may include collaboration with participants’ employers. The primary outcome sickness absence is operationalised as the number and proportion of individuals who remain in full- or part-time work, the number of gross and net days of sickness absence during the intervention and follow-up period, and time to first sickness absence spell. Secondary outcomes are patient-reported short-term sickness absence, work ability, pain, self-efficacy, health-related quality of life, risk for sickness absence, anxiety and depression symptoms and physical disability at 1 and 3 months after inclusion (short-term follow-up), and at 6 and 12 months (long-term follow-up). A cost-effectiveness analysis is planned and drug consumption will be investigated.

**Discussion:**

The study is expected to provide new knowledge on the effectiveness of a comprehensive rehabilitation model that incorporates early identification of patients with musculoskeletal pain at risk for development of sickness absence and persistent pain. The study findings may contribute to more effective rehabilitation processes of this large patient population, and potentially reduce sickness absence and costs.

**Trial registration:**

ClinicalTrials.gov Protocol ID: NCT03913325, Registered April 12, 2019.

Version 2, 10 July 2020.

Version 2 changes: Clarifications regarding trial aim and inclusion process.

## Background

The increasing burden from musculoskeletal disorders has been shown in the 20-year follow-up of the Global Burden of Disease Study (GBDS) and, even though musculoskeletal pain rarely is fatal, it threatens peoples’ health and account for a large amount of disability adjusted life years (DALYs) [1]. Approximately 10% of individuals who suffer from musculoskeletal pain develop disabling persistent problems, and, as such, are at increased risk of long-term sickness absence [2]. In Sweden, almost half of all ongoing sickness benefits reimbursed by The Swedish Social Insurance Agency (SSIA) relate to musculoskeletal disorders and mild to moderate mental illnesses [3]. Even though there has been a focus on return to work for many years in Sweden, yielding governmental support, recent years have seen an increase in sickness absence. Between 2010 and 2016 sickness cash benefits increased by 80% [4]. Musculoskeletal conditions accounted for 19% of all sick-leave cases among women and 24% of all cases among men, and were more common in people older than 50 years. However, in the beginning of 2017 a decrease was seen [4]. Persistent pain and reduced work capacity have been shown to be strongly associated, and the probability for disability pension or unemployment is significantly higher for individuals with persistent pain [5]. Subsequently, there is increasing consensus that there is a need for early identification and prevention of the development of persistent pain, and to prevent reduced work ability that may lead to sickness absence.

Musculoskeletal pain is also a common reason for consulting primary health care [6], at substantial costs, creating a demand on the healthcare system to provide accurate and timely diagnosis and treatment. This underscores the need for early identification of individuals at risk of developing persistent pain, which might not only require prolonged contact with healthcare services but potentially lead to sickness absence due to reduced work ability.

A systematic review from The Swedish Health Technology Assessment [7] aimed to provide evidence-based practice recommendations for interventions in the acute and subacute phase of neck and back pain, preventive for persistent pain. However, it was not possible to draw such conclusions as the included studies compared different combinations of interventions in a way that made it unclear which of the measures taken were actually preventive. The review concluded that more rigorous and scientifically sound studies were needed.

Nevertheless, psychosocial factors, independently as well as in interaction with physical factors, have been shown to be associated with risk of sickness absence [8], and a biopsychosocial approach using interdisciplinary teamwork seems well suited for conditions that already are, or are at risk of becoming, chronic [9, 10].

A person-centred approach and shared decision making are widely advocated as key components of effective illness management [11]. Even though more research is needed, it seems that a person-centred approach could positively affect rehabilitation outcomes [12]. It has been shown to improve self-efficacy in patients with acute coronary syndrome when used throughout the chain of care from hospital to primary care [13] and also to improve health-related quality of life when used in a prehabilitation program for patients scheduled for lumbar fusion surgery [14].

An important factor shown to be related to better functioning outcome in patients with persistent pain is high self-efficacy [15]. Self-efficacy refers to the confidence that one has in one’s capabilities to successfully execute a course of action to a desired outcome [15]. Patients with musculoskeletal disorders have reported a preference for participating and sharing clinical decision making with their rehabilitation professional, and participating in clinical decision making has been suggested to lead to the patient’s active engagement in their rehabilitation [16]. Patients with a higher degree of personal responsibility were more likely to report a better outcome of physiotherapy treatment [17]. However, it also has been shown that peoples’ willingness to take responsibility for the management of musculoskeletal pain should not be underestimated. Patients have described how the responsibility should be shared with the medical professionals but also identified and met by society, employers and family [18].

Poor communication and information exchange have been reported as important barriers for effective collaboration among different health and welfare services [19]. It has been reported that the collaboration between health care and employers is to be difficult to achieve, that the relationships with the social insurance officers is often strained, and this is affecting collaboration among the three parts [20]. A systematic review [21] on the effectiveness of workplace interventions in return to work for musculoskeletal pain recommended implementing multi-domain interventions (i.e. with healthcare provision, service coordination, and work accommodation components). The planned study will draw on these findings to establish responsibilities, clear roles, and routines for communication and collaboration among the patient, employer and other stakeholders.

The use of cognitive behavioural therapy (CBT) based strategies in physiotherapy interventions has been shown to be beneficial for preventing persistent low back pain [22]. Adding a psychological intervention (generally CBT) to physiotherapy for patients with persistent pain has been shown in a systematic review and meta-analysis to significantly improve physical function and quality of life compared with physiotherapy alone [23]. The psychological component was generally delivered by clinical psychologists, implying that their training and skill set likely is necessary to achieve the desired outcome. Many other studies also have contributed to establishing a solid evidence base for CBT as treatment for persistent pain, suggesting that it also would be beneficial for those at risk of developing persistent pain [24, 25]. Hence, there still is a gap in knowledge on how to prevent the development of persistent musculoskeletal pain and the subsequent limitations in daily life. The planned study will include early access to psychological intervention.

To conclude, sickness absence due to persistent musculoskeletal pain is complex; therefore a rehabilitation model was designed to provide an early intervention for prevention of sickness absence for musculoskeletal pain, called the PREVSAM model. The model incorporates a person-centred approach in a biopsychosocial model using interdisciplinary teamwork and possible collaboration with the workplace, where clear assignments of who is responsible for what actions are set. The model will be evaluated in a randomised controlled trial.

### Aims

The aim of the study is to evaluate effects of an early intervention, the PREVSAM model, on the prevention of sickness absence and development of persistent pain in at-risk patients with musculoskeletal pain. The study will also evaluate effects of the PREVSAM model on patient-reported work ability, risk for sickness absence, musculoskeletal pain status, self-efficacy, health-related quality of life, physical disability, depression and anxiety symptoms, and drug consumption. Cost-effectiveness of the model will be evaluated.

Research questions:

Q1: Is the PREVSAM model more effective in preventing sickness absence, compared with treatment as usual, in patients with musculoskeletal pain who have been identified to be at risk for sickness absence?

Q2: Do patients participating in the PREVSAM model report higher work ability, improved pain status, better self-efficacy, less physical disability or higher health-related quality of life, compared with those who receive treatment as usual at follow-up?

Q3: Is there a patient-reported reduced risk (using the ÖMPSQ score) for sickness absence in patients participating in the PREVSAM model, compared with those who received treatment as usual at follow-up?

Q4: Can any effects be seen in regard to anxiety and depression symptoms or drug consumption in patients participating in the PREVSAM model, compared with those who receive treatment as usual?

Q5: Is the PREVSAM model more cost-effective compared with treatment as usual?

The study hypothesis is that a person-centred rehabilitation, illuminating and emphasizing responsibilities, using interdisciplinary teamwork which can also include collaboration with the employer and/or workplace, in an early stage before symptoms are manifest and consequences in form of sickness absence have become established, will be more effective than treatment as usual in prevention of sickness absence.

## Methods

This randomised controlled trial has been registered with ClinicalTrials.gov, Protocol ID: NCT03913325, initial release 12/04/2019.

### Study design

**Fig. 1:**
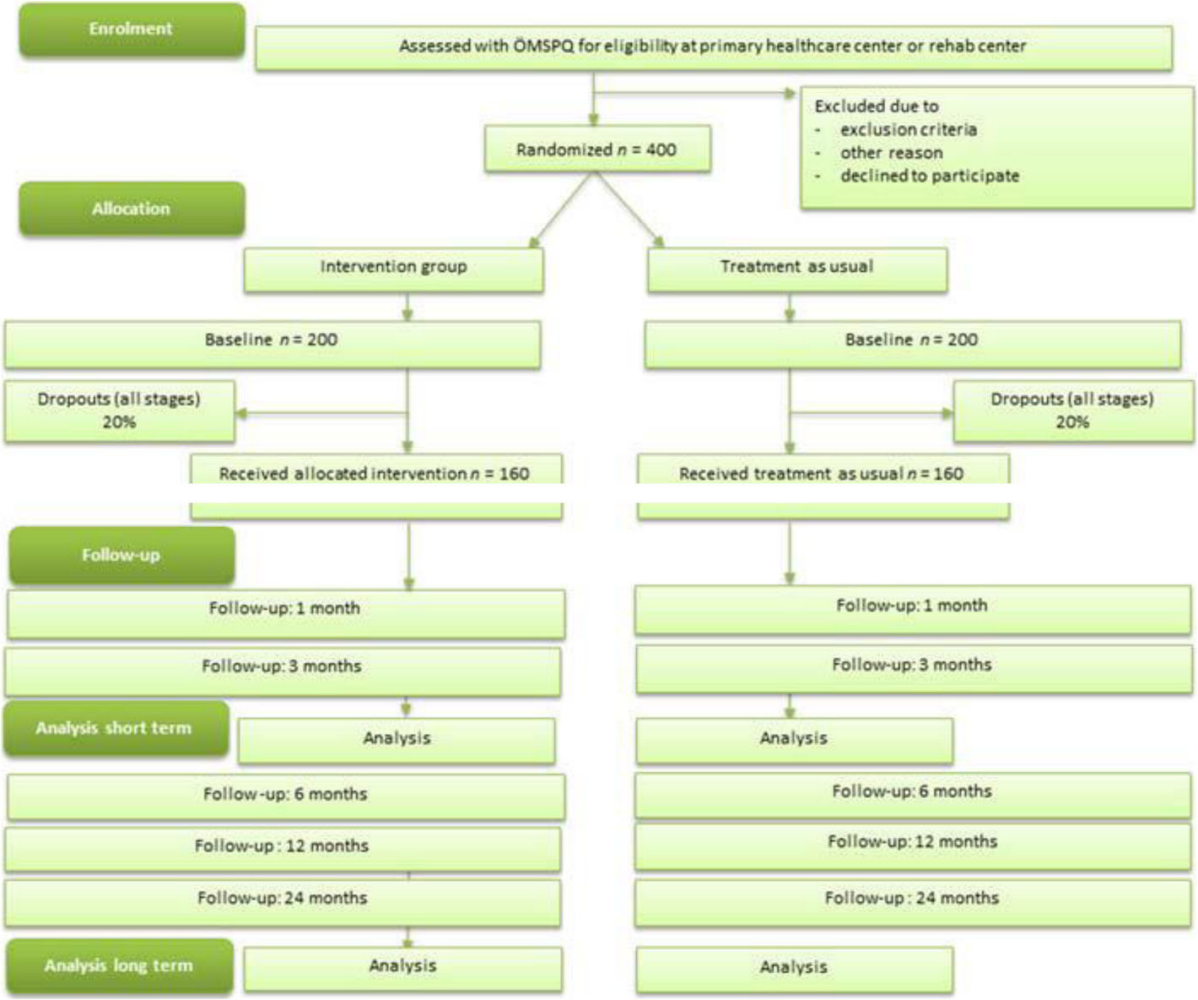
Flow chart of participants through the study

A randomised, controlled, two-armed, superiority trial will be conducted, in which treatment according to the PREVSAM model (intervention group) is compared with treatment as usual (TAU) (control group). Findings will be reported according to the CONSORT Checklist. The study design is illustrated in Fig. 1.

### Study population and setting

Adult individuals in Region Västra Götaland who seek health care for musculoskeletal pain at primary care rehabilitation centres or primary care healthcare centres are potentially eligible for inclusion. A list of study centres will be available from the corresponding author on request. Inclusion and exclusion criteria for study participation are described in Table 1.

**Table 1:** Inclusion and exclusion criteria for study participation

*Inclusion criteria*	*Exclusion criteria*
Aged ≥18 years	Pain not primarily generated from the musculoskeletal system
Understand Swedish sufficiently to be able to fill out the questionnaires	Sickness cash benefit more than 30 days during the last 12 months due to musculoskeletal pain
Having a paid job or receiving parental leave benefits, unemployment benefits or student aid	Full disability pension or retired due to age
Musculoskeletal pain for less than 3 months during the current episode	Pregnancy
Risk for development of persistent musculoskeletal pain and disability, indicated by a score ≥ 40 on the ÖMPSQ-SF	Red flag disorders such as malignancy/ cancer, acute major traumas such as multiple fractures (less than 6 months ago) or infection, spinal cord compression/cauda equina
Independently mobile (with or without aids), to be capable of participating in intervention	Severe mental illness, ongoing substance abuse disorders

For inclusion, participants will be screened using the Örebro Musculoskeletal Pain Screening Questionnaire Short Form (ÖMPSQ-SF) [26]. A total score below 40 points on the ÖMPSQ-SF indicates a low risk for development of manifest problems and sickness absence, while scores over 40 points indicate an increased risk. In the screening process we will select individuals with ÖMPSQ-SF score equal to or greater than 40.

### Recruitment

All public rehabilitation centres and a majority of the private rehabilitation centres in the Region Västra Götaland’s Care choice primary care, have been informed about the study and invited to participate. We expect approximately 10 rehabilitation centres to be interested in participating and, during 2019, we have established contact with the participating centres and provided the necessary infrastructure for support of patient recruitment and intervention. To achieve adequate participant enrolment and reach the target sample size, additional centres may be added (from primary care rehabilitation centres or primary care healthcare centres). Interdisciplinary teams are formed by healthcare professionals at the participating centre or can be complemented by a consultant.

Patients at the participating centres will be screened for eligibility. Those fulfilling the inclusion criteria will be provided with oral and written information before asked to participate. Those accepting participation will complete a written informed consent form.

### Randomisation

The participants will be randomised to either the PREVSAM model or to treatment as usual (TAU). Sequentially numbered, opaque, sealed envelopes containing notes marked ‘PREVSAM’ or ‘TAU’ are block-randomised by a computer programme in blocks of six (three of each), in order to ensure an even distribution. These are placed in a box at each participating centre. The allocation sequence will not be accessible to the persons who enrol participants and assign intervention. Enrolment and assignment of intervention will most often be done by a person from the interdisciplinary team.

### Blinding

Due to the nature of the intervention, neither participating healthcare professionals nor patients will be blinded to treatment. Investigators, outcome assessors and data analysts will be blinded to group allocation.

### Intervention: the PREVSAM model (intervention group)

The PREVSAM model includes:

- a person-centred approach
- identification of the patient’s attitudes towards responsibility for the management of musculoskeletal pain and clarification of responsibilities for the individual, health care, employer/Swedish public employer service
- structured, individualised, synchronised, rehabilitation based on interdisciplinary teamwork, continuous communication and regular coordination among all stakeholders, and a mutual philosophy of rehabilitation
- early access to psychologist or psychotherapist to address psychosocial factors
- possibility of early contact and collaboration with employer/workplace before sickness absence has occurred.

The person-centred approach entails planning the rehabilitation together in partnership with the individuals, and taking their resources and wishes into consideration [27]. Individualised, person-centred, rehabilitation within the PREVSAM model will be based on the patient’s narrative and constructed together with the interdisciplinary team based on the patient’s goals, attitudes of responsibility, and physical, psychological, and social resources. Workplace preconditions will be assessed, preferably based on the Convergence Dialogue Meeting [28] also used in a previous rehabilitation study [29], and employer responsibility will be highlighted, based on the Organisational and social work environment provisions from the Swedish Work Environment Authority (AFS 2015:4). Responsibilities for the different parts of the participant’s rehabilitation will be clearly assigned to the individual, health care, and employer or Swedish public employer service.

The interdisciplinary team is an important component of the PREVSAM model and will be comprised of the patient, a physiotherapist, an occupational therapist, and a psychologist/psychotherapist. If needed, contact with a general practitioner will be provided. If orthopaedic consultation or further investigations are needed, the participant can be referred to a physiotherapist with experience in orthopaedic triage for assessment and further management.

The PREVSAM model can also reach outside the healthcare system. Contacts can be taken immediately to facilitate collaboration to avoid sickness absence, usually with the employer/workplace, but could also involve the Swedish Public Employment Agency and the SSIA. Furthermore, a rehabilitation coordinator could be contacted, if necessary and available. This function within the healthcare system is specifically directed at internal coordination, external collaboration and individual patient support in questions related to sickness absence. In the PREVSAM model, adjustments of work parameters will be given early attention if there are work-related issues, rather than waiting for the patient to “get better” first. The individual rehabilitation should be planned to avoid a need for the patient to be on sickness absence (with the exception of preventive sickness cash benefit), as it has been suggested this might have a lock-in effect and increase the risk of long-term sickness absence [30]. Because each PREVSAM rehabilitation plan is individualised, the length of the rehabilitation period will differ for each individual, but will in most cases not exceed 8 weeks. If considered appropriate by the team and the patient, follow-up with the employer can take place.

Physiotherapists, occupational therapists and psychologists or psychotherapists will receive education, training and instructions in using the PREVSAM model at their workplace by the project coordinator before starting the study. They will also participate in workshops while the study is ongoing, together with the involved psychotherapist. The workshops aim to improve the teamwork, enable the practicing of the core values of the model, and provide discussions on possible ambiguities and difficulties, as well as sharing good experiences. They are expected to ensure adherence to the intervention protocol. The project coordinator will have regular contact with the participating centres through a monthly newsletter, e-mail or telephone on any practical or methodological questions.

### Comparison: treatment as usual (control group)

The participants randomised to TAU will receive management and treatment as usual, according to the choice of the responsible healthcare professional(s). Treatments may include information and advice, physical exercise, functional and ergonomic training, mobilisation/manipulation, relaxation, body awareness training, acupuncture, activity and pacing, stress management, lifestyle issue, etc. Treatment as usual was chosen as comparator because that is what the patient group is provided as best choice of treatment of today, and the study aims to evaluate whether a more comprehensive rehabilitation model would result in superior results. The type of consultation and interventions provided will be collected from the participant’s electronic medical record. Any other healthcare measures provided for the same diagnosis will be collected from the Region Västra Götaland’s healthcare utilisation database (VEGA).

The main components of the intervention and the comparator are summarised in Table 2.

**Table 2:** PREVSAM model compared with treatment as usual

*PREVSAM model*	*TAU*
Medical history with a person-centred approach and structure	Medical history could include a person-centred approach
Physical examination	Physical examination
Identification and clarification of the patient’s attitudes towards responsibility for the management of musculoskeletal pain	
A rehabilitation plan with structured, individualised, synchronised rehabilitation based on interdisciplinary teamwork.	Individualised rehabilitation plan
Possibility of early access to psychologist or psychotherapist	Could be possible to see psychologist or psychotherapist
Possibility of early contact and collaboration with employer and workplace	

TAU = treatment as usual

### Outcomes

#### Primary outcome

##### Sickness absence

Sickness absence will be measured using the proposed outcome measures from the Swedish Social insurance report 2016:9 [31]. These measures are based on sick-leave data at the individual level (including diagnoses) of all sick-leave spells longer than 14 days in which sickness cash benefits were provided by the SSIA. Data will be retrieved from the MiDAS database (preventive sickness absence will be excluded as this might be part of intervention). All sickness absence is planned to be analysed, but the main focus is on sickness absence relevant to musculoskeletal pain. Sickness absence was chosen as primary outcome because it is a frequent consequence of musculoskeletal pain, and it is of relevance for both the individual, the employer and society to measure and target this outcome when testing and implementing a new rehabilitation model for this patient group.

Sickness absence will be operationalised as:

1. Number and proportion of individuals who were on sickness absence and/or the number and proportion of individuals who remain in full- or part-time work during the follow-up period.
2. Number of gross and net sick-days during the follow-up period.
3. Time to first sickness absence spell.

Short-term sickness absence (≤ 14 days) is not reported to the SSIA among employed participants. They have sick pay from their employer the first 2 weeks of a sick-leave spell. Thus, short-term sickness absence will be measured using self-reported responses to weekly text messages via mobile phone (SMS-Track ApS, Denmark, www.sms-track.com). The participants will enter a number from 0 to 7 in response to the question “Have you been absent from work during the past week due to your musculoskeletal pain, and if so for how many days?”. However, unemployed participants have sickness benefits from the SSIA from the second day of a sick-leave spell.

#### Secondary outcomes

##### Patient-reported work ability

As registered sickness cash benefit for sick-leave not always is equivalent to the self-perceived ability to work, a single-item question of the Work Ability Index (WAI) [32], “current work ability compared with the lifetime best” will also be used. This question has been found to correspond well to WAI categories of the full instrument [33].

##### Patient-reported pain status

*Pain frequency* during the previous week will be measured using a 4-point Likert scale from 1 (always/nearly always) to 4 (rarely). *Pain duration* (0–1 week (w), 2–3 w, 4–5 w, 6–7 w, 8–9 w, 10-11w, 12–23 w, 24–35 w, 36–52 w or more than 52 w) will be registered. *Pain intensity* during the previous 7 days will be measured using an 11-point numeric rating scale (NRS) [34] anchored by “no pain at all” and “worst imaginable pain”). The NRS has been shown to have better responsiveness, ease of use, compliance rate, and applicability than a visual analogue scale and a verbal rating scale [35]. *Number of pain sites* the previous 7 days will be measured using a predefined manikin where the patient marks the location of their pain on an anatomical sketch of a human body.

##### Patient-reported self-efficacy

A 2-item short form of the Pain Self-Efficacy Questionnaire (PSEQ-2) will be used to measure changes of beliefs held regarding the possibility to carry out certain activities even when experiencing pain. The PSEQ-2 is a short form of the Pain Self- Efficacy Scale (PSEQ), which is a well-established and widely used measure of self-efficacy in people with chronic pain [36]. The PSEQ-2 has evidence of validity and reliability in people experiencing chronic pain [37]. Items 5 and 9 from the PSEQ are used in PSEQ-2. PSEQ-2 will be translated into Swedish before use, following the procedure described by Beaton et al. [38]. One informed and one uninformed Swedish native translator will translate the two items, response options, and questionnaire instructions. They will do a written report about difficulties and challenges with the translation, compare their versions, and merge their translations into a synthesised version. An observer will notice how they reach consensus. A synthesised translation will be back translated into English by two English native translators, and a written report will be made for each version. An expert committee will read the reports and the two versions, and discuss to reach consensus and produce a final Swedish version of the questionnaire [38].

The General Self-Efficacy Scale (GES) will be used to assess the strength of an individual’s belief in his/her own ability to respond to difficult situations and to execute a course of action to a desired outcome [39]. It consists of 10 items rated on a 4-point Likert scale (“not at all true” to” exactly true”). The Swedish translation of the GES has proven to be a reliable indicator of perceived general self-efficacy [40].

##### Patient-reported health-related quality of life

The European Quality of Life-5 Dimensions Questionnaire (EQ-5D-5L) will be used to assess health-related quality of life [41]. The EQ-5D has evidence of validity and reliability in different musculoskeletal disorders [42].

##### Patient-reported risk for sickness absence

The ÖMPSQ-SF will be used primarily as a screening instrument for the planned study [26], but also as an outcome measure for *changed risk* (proportion lowering their risk by scoring < 40 p at follow-up). The original Örebro Musculoskeletal Pain Screening Questionnaire (ÖMPSQ) was developed as a screening questionnaire for identifying patients at risk of developing persistent back pain problems, and has been shown to have acceptable reliability and validity [43]. It is often described as predictive for persistent pain, but studies have usually evaluated the predictive validity of the questionnaire for sickness absence [26, 43]. The ÖMPSQ-SF is self-administered and includes item 7, 9, 13–16, 19–21 and 25 from the original ÖMPSQ. The item contains a statement or an assertion which the respondents rate on 11-point Likert scales from 0, referring to absence of impairment, to 10, severe impairment. A cut-off of 50 points has been recommended to identify those of higher risk of future disability and sickness absence [26]. However, clinical experience as well as recent studies have shown that a cut off of 40 points might be better to use [29]. Although the instrument was primarily developed for people with back pain, the questions regarding musculoskeletal pain are general and can be used for other regions of musculoskeletal pain. The short version has been shown to be appropriate for clinical and research purposes, since it is nearly as accurate as the original, longer version [26].

##### Patient-reported anxiety and depression symptoms

Assessed with the Hospital Anxiety and Depression scale (HADS). The HADS is a 14-item measure of self-reported symptoms of anxiety (7 items) and depression (7 items) [44]. Each item is scored from 0 to 3, indicating absence of symptoms or presence of positive features (0) to maximal presentation of symptoms or the absence of positive features [3]. The HADS has been validated and found to have good psychometric properties [45, 46].

##### Patient-reported physical disability

Assessed with the Disability Rating Index (DRI). The DRI is a self-assessment measure of experienced physical disability by patients with musculoskeletal disorders. It includes twelve consciously unspecified everyday activities that the vast majority of people perform or can imagine. The questions are arranged in order of increasing physical demands, where the participants mark on a 100-mm visual analogue scale (VAS) in accordance to her/his presumed ability to perform the daily physical activities in question. The anchor points are “without difficulty” = 0 and “not at all” = 100. The instrument has good reliability, validity and responsiveness [3].

##### Drug consumption

Consumption of pharmaceutical drugs that can be related to the musculoskeletal condition for which the participant is treated (ATC codes M – musculoskeletal system and N – nervous system) will be estimated using data from the Swedish registry for drug use, regarding filled prescriptions. Data will be collected for a 3-month period before baseline and for the 12-month period after baseline. Self-reported drug consumption, both prescription and nonprescription drugs, will be collected via the questionnaire.

Other secondary outcome measures are loss of productivity and healthcare costs and incremental cost-effectiveness ratios [17].

### Data collection

For the primary outcome sickness absence, data will be collected from the SSIA and self-reported short-term sickness absence will be collected weekly from the participants as described above.

For the secondary outcomes, an email is sent to the patient with a link to the questionnaire where the patient-reported data will be collected using the self-administrated instruments at inclusion (baseline), at one and 3 months after inclusion (short-term follow-up), and at six and 12 months after inclusion (long-term follow-up). Data on age, gender, location of birth, education, family, present employment or source of income, and symptom duration will be collected at inclusion. The patient drawing of the pain sites marked on an anatomical sketch of a human body is sent out separately by mail and returned in a pre-paid envelope. A reminder is sent by email once per week during 4 weeks, to promote participant retention and complete follow-ups. For the patient drawing, one reminder is sent out after 2–3 weeks by mail. If a patient wishes to withdraw from the study or further follow-ups, he or she is asked to approve the collection of registry data from SSIA and the Swedish registry for drug consumption.

Data on interventions received in both the PREVSAM and the TAU group will be collected from the participant’s electronic medical record, which also will be used for monitoring of intervention adherence/treatment fidelity. The interdisciplinary teams are instructed to report on solicited and spontaneously reported adverse events and other unintended effects of trial interventions or trial conduct. Any concomitant interventions provided for the same diagnosis will be collected via Region Västra Götaland’s healthcare utilisation database (VEGA).

Outcome data from the web-based questionnaire will be transferred to a database, where it will be checked for accuracy by the research assistant to promote data quality.

There will be no data monitoring committee since the study is independent from the sponsor and the intervention consists of a rehabilitation model that includes evidence-based treatment methods with little risk for unexpected adverse events.

### Statistical analyses

Primary outcomes are sickness absence and sick days, where the first outcome is binary and the second one is continuous and most likely skewed [47]. Sick leave regardless of reason will be retrieved. However, sick leave for diseases of the musculoskeletal system and connective tissue is of specific interest. Sample size calculations will be based on the general assumptions that we want a power of 80% and that statistical significance is set to α = 0.05. For the outcome sickness absence, the difference between the proportion of sickness absence = yes/no will be compared between the intervention (PREVSAM model) and control (TAU) group. We assume that approximately 30% in the control group will have sickness absence = yes at least one time during the 12 month follow-up period [48]. If the percentage of sickness absence = yes in the intervention group is assumed to be 15% or 20%, then the sample size in each group needs to be 312 individuals and 132 individuals, respectively. To detect a difference of 15% (30% with sickness absence in control group and 15% with sickness absence in intervention group), a total sample size of 264 (132 individuals in each group) would give approximately 80 cases to analyse for the primary outcome sick days. This would enable us to estimate 8 parameters in a logistic regression analysis. This would mean a power of about 75% to detect a difference of at least 0.6 Ln (sick days), if the standard deviation for Ln (sick days) is assumed to be 1. This is equal to a scenario with mean sick days of about 35 in the intervention group (PREVSAM model) and 68 in the control group (TAU). This would enable us to perform linear regression analysis adjusting for several covariates and estimating about 7 parameters. To summarise these calculations and allowing for dropouts, randomisation of 200 individuals to each group is required. An interim analysis will be performed for the primary outcome when 50% of patients have been randomised and have completed the 12 months follow-up. At the same time-point it might be possible to perform the short-term follow-up analyses. The interim analysis will be performed by a statistician, blinded to the treatment allocation. The statistician will report and discuss the results of the interim analysis with the steering committee in a joint meeting. The steering committee decides on the continuation of the trial.

Group characteristics will be presented as mean and standard deviations, median and 25th and 75th percentile or the number and percentage. For the primary outcomes descriptive analyses for sickness absence for the two groups will be presented according to recommendations [31]. The sickness absence will be presented with number of individuals who had sickness absence during the follow-up period. Proportions of individuals with sickness absence per month will be presented. The number of sick days (gross and net) with sickness benefit during the follow-up periods will be presented with mean and median values. For participation in work life additional measures will be presented with descriptive statistics regarding time to sick-leave spell for musculoskeletal pain. Number of days before a sick leave spell > 14 days occurs and number and duration of sick-leave spells per individual will be presented for gross and net days. Outcomes will be analysed according to intention-to-treat. Multivariable logistic regression analysis will be used for the primary outcome sickness absence during the follow-up period [1], not being sickness absent (0) adjusted for confounders. Multivariable linear regression analyses, or possibly quantile regression, will be used to evaluate differences between groups for sick leave days, among those have at least one sick leave spell. All other measurements will be analysed by calculating raw differences between baseline and follow-up assessments. Parametric and non-parametric tests will be used depending on data level and distribution. Sub-group analyses can be performed in regard to characteristics of interest, such as diagnosis or sick-leave. The level of significance is set to *p* < 0.05. Relevant statistical programs will be used for the statistical analyses, for example the IBM SPSS, SAS, STATA.

### Ethical considerations and data management

Even though the intervention does not include any new technology per se, but rather a more comprehensive, yet focused, rehabilitation model, several ethical issues need to be considered. Screening for a condition can sometimes be controversial. There might be a risk that we find too many cases and “overtreat” these people. However, if the study does not find any benefits of the new model, we will have a clear indication that there is no need to introduce this more comprehensive model at an early stage.

There is also a possibility that people are hesitant about healthcare professionals approaching and collaborating with their employer. However, clinical experience has shown that people often perceive that the healthcare system can be a legitimate channel to reach the employer to explain the needs, and also to stress the responsibilities of each part.

Eligible participants who meet the inclusion criteria and preliminary agree to participate, will receive oral and written information, including the written consent form, by a healthcare professional at the study centre, before or at their first visit to the clinic. The information includes that participation is voluntary and that they may withdraw participation at any time, without any consequences for their continued treatment or rehabilitation. If the patient accepts participation, he/she returns the signed informed consent to the study centre and these are collected by the project coordinator. The informed consent can also be sent back by mail to the project coordinator. In the informed consent the participants also consent to collection of registry data.

The study will involve collection and management of personal demographic and outcome data, which will be treated with high respect for the persons’ integrity. All collected data will be coded and a code list will be created, giving the possibility to go back and double check raw data. Data and code list will be stored separately and will only be accessible by authorised members of the research team, as will the final trial dataset. Data will be managed according to Swedish law and in compliance with Region Västra Götaland’s data management guidelines. This includes registry with the Data Protection Officer of Region Västra Götaland and storage in a locked and fireproof safe and on access-controlled servers with high levels of security.

A data management plan (DMP) has been developed, which provides details on how data are collected, managed during and after the project, documented, stored and archived, and made available, including, where, when, how and to whom. The DMP can be provided on reasonable request from the corresponding author. Routines for data storage from Region Västra Götaland will be followed and meta data standards will be used to describe the data material. A data management team will manage and monitor data accuracy and quality, and comprises the principal investigator, a postdoc or senior researcher, a project coordinator, and a research assistant.

### Timeline

**Fig. 2:**
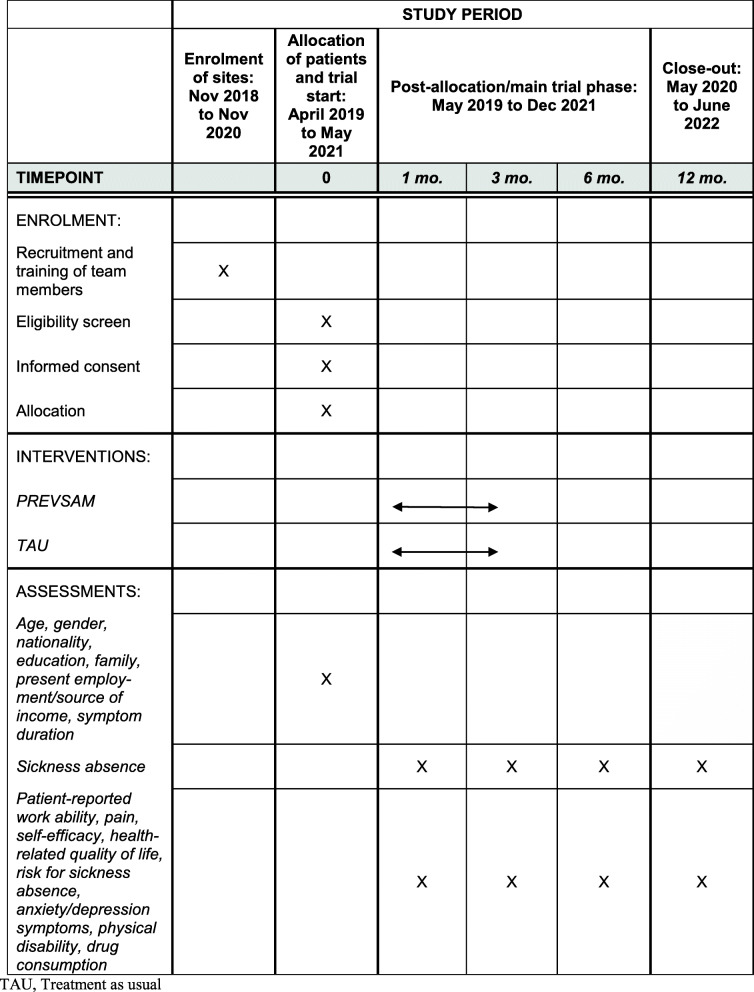
Schedule of enrolment, interventions, and assessments. TAU, Treatment as usual

A timeline and schedule of enrolment, interventions, and assessment is presented in Fig. 2.

### Process evaluation

In parallel to the intervention, a process evaluation is planned, in which implementation and process outcomes will be monitored, documented, and assessed [49]. All interventions delivered will be documented and closely monitored to enable evaluation and further development of the rehabilitation model, in which issues such as detailed intervention components, treatment/model fidelity, and training will be addressed. The process evaluation will facilitate future implementation and scale-up of the intervention, should it be found effective in this study.

### Dissemination and implementation of study findings

This study protocol will be published in an open access journal to allow free public access. The study findings will be disseminated and made publicly available in peer-reviewed publications and conference presentations. The results will also be disseminated, through regular dissemination and communication channels, within and outside health care and university contexts. The Swedish research Council for Health, Working Life and Welfare that sponsors the study usually organises conferences to disseminate results from studies they sponsor. Since the planned study focuses on collaboration both within health care and with employers and the SSIA, communication strategies targeting these stakeholders will be developed.

## Discussion

This randomised controlled trial will evaluate the effects of a new rehabilitation model that comprises interdisciplinary, person-centred interventions for individuals with musculoskeletal pain who are at risk for persistent pain, physical disability and sick leave. Effects on sickness absence and various patient-reported health outcomes will be measured. The study is expected to show whether a rehabilitation model of this nature can be a feasible method in a primary care context to prevent or reduce sickness absence due to musculoskeletal pain.

The pragmatic nature of the PREVSAM model and its closeness to current clinical practice provide a clinical advantage and will make the intervention easy to implement throughout the county and beyond. The study will provide new knowledge on if identifying patients with musculoskeletal pain at risk for sickness absence and development of persistent pain, and providing this structured, person-centred, interdisciplinary rehabilitation is more effective than treatment as usual. The long-term implications of the study are to optimise rehabilitation for this large patient group, which may through secondary prevention of pain help reducing sick leave and improving return to work rates and participation in society, as well as reduce costs for both healthcare and society.

Year 2008, multimodal rehabilitation (MMR) programmes were initiated with governmental support in the form of “Rehabilitation guarantee”, but neither evaluations in Region Västra Götaland nor other regional or national evaluations found any significant reductions in sickness absence [50–52]. The PREVSAM model is designed to be feasible to perform within the existing “Care choice rehabilitation of primary care” context in the Region Västra Götaland, if showed to be effective, successful implementation will probably require adjustments in the reimbursement system; e.g. reimbursement will be required also for psychological interventions within rehabilitation team meetings, as well as meetings and activities at the workplace. There has been an ongoing debate for at least 25 years whether health care should be performing actions at or in collaboration with the workplace; however, interventions including healthcare provision in combination with service coordination and work accommodation have been recommended to improve work functioning and reduce costs [21]. Although there is general consensus that health care has the competence of doing so, the discussion is still about reimbursements. A recent study showed that workplace dialogue in addition to physiotherapy improved work ability significantly in patients with acute/subacute neck and/or back pain [29], and that the combination of workplace dialogue and physiotherapy was cost-effective from both a societal and a healthcare perspective [53]. It has also been shown that occupational therapy interventions increase return to work rates [54] and, that cognitive behavioural therapies showed positive results on return to work and sick leave for people with chronic illnesses [55]. In this study the physiotherapist, the occupational therapist, and the psychotherapist constitute the core interdisciplinary team.

The costs for the PREVSAM rehabilitation model described in this protocol, if implemented on a large scale, are expected to be modest and the intervention is likely to yield similar cost-effectiveness when treatment costs are weighed with the indirect costs of sickness absence.

Because the return-to-work process for individuals who are sick-listed is costly for both individuals, health care and society, it is of great importance to find ways of preventing sickness absence and of keeping acute or subacute musculoskeletal pain from becoming persistent. Our study is expected to provide new knowledge regarding the effectiveness of a rehabilitation model that may be one such way, and its feasibility in a primary care context.

### Limitations and strengths

The main limitation of the trial is that neither patients nor caregivers will be blinded. This is not feasible due to the nature of the intervention. The multicentre nature of the study, involving multiple intervening therapists in multiple locations, as well as the individualised, person-centred intervention approach, entails a risk of the interventions being implemented with suboptimal fidelity. However, the included training and detailed written intervention protocols are designed to minimise this risk.

A strength of the study is that the rehabilitation model includes interventions already tried and proven, and used in clinical practice. This, together with the very fact that it is a multicentre trial, may strengthen generalisability of the programme.

## Data Availability

Not applicable as there is no existing data set yet.

## Abbreviations

AF: Public employment service
ARM: Attitudes regarding Responsibility for Musculoskeletal disorders
DRI: Disability Rating Index
EII: Early Identification and Intervention
EQ-5D: European Quality of Life-5 dimensions questionnaire
GBDS: Global Burden of Disease Study
HADS: Hospital Anxiety and Depression Scale
MMR: Multimodal rehabilitation
PHCC: Primary healthcare centre
PREVSAM: **PREV**ention of **S**ickness **A**bsence due to **M**usculoskeletal pain
PSEQ: Pain Self-Efficacy Questionnaire
PSEQ-2: Pain Self-Efficacy Questionnaire – short form
RCT: Randomised controlled trial
SEK: Swedish kronor (crowns)
SSIA: Swedish Social Insurance Agency
TAU: Treatment as usual
ÖMPSQ: Örebro Musculoskeletal Pain Screening Questionnaire
ÖMPSQ-SF: Örebro Musculoskeletal Pain Screening Questionnaire – Short Form
WAI: Work Ability Index

## References

[1] Murray CJ, Atkinson C, Bhalla K, Birbeck G, Burstein R, Chou D (2013). The state of US health, 1990-2010: burden of diseases, injuries, and risk factors. JAMA..

[2] Airaksinen O, Brox JI, Cedraschi C, Hildebrandt J, Klaber-Moffett J, Kovacs F (2006). Chapter 4. European guidelines for the management of chronic nonspecific low back pain. Eur Spine J.

[3] Salen BA, Spangfort EV, Nygren AL, Nordemar R (1994). The disability rating index: an instrument for the assessment of disability in clinical settings. J Clin Epidemiol.

[4] Försäkringskassan (The Swedish Social Insurance Agency). Sjukpenningtalet i november 2017. Press release Dec 22, 2017 [Available from: https://www.forsakringskassan.se/press/pressmeddelanden/!ut/p/z0/fYzLCsIwEEW_pssyY7Gp2y7UUhEFQWs2JZqhpI9J2sTi51vEtatzD1wOSKhAsppNo4KxrPrF71LUm3OxLkpMjnjIt5hnV7FDcUpEinAhhhLk_9NSMe04yhzk03Kgd4DqOzh4qifyzrI3M0XoFvE_DKQ19Yo1cYS-fXWOmA03PqieQmxitjMND5riBFcZuG5_-wB8NBJ4/?keepNavState=true.].

[5] Landmark T, Romundstad P, Dale O, Borchgrevink PC, Vatten L, Kaasa S (2013). Chronic pain: one year prevalence and associated characteristics (the HUNT pain study). Scand J Pain.

[6] MacKay C, Canizares M, Davis AM, Badley EM (2010). Health care utilization for musculoskeletal disorders. Arthritis Care Res.

[7] SBU. Preventiva insatser vid akut smärta från rygg och nacke (2016). En systematisk översikt och utvärdering av medicinska, hälsoekonomiska och etiska aspekter.

[8] Bergstrom G, Hagberg J, Busch H, Jensen I, Bjorklund C (2014). Prediction of sickness absenteeism, disability pension and sickness presenteeism among employees with back pain. J Occup Rehabil.

[9] Gatchel RJ, Peng YB, Peters ML, Fuchs PN, Turk DC (2007). The biopsychosocial approach to chronic pain: scientific advances and future directions. Psychol Bull.

[10] Gatchel RJ, McGeary DD, McGeary CA, Lippe B (2014). Interdisciplinary chronic pain management: past, present, and future. Am Psychol.

[11] Morgan S, Yoder LH (2012). A concept analysis of person-centered care. J Holist Nurs.

[12] Yun D, Choi J (2019). Person-centered rehabilitation care and outcomes: a systematic literature review. Int J Nurs Stud.

[13] Fors A, Ekman I, Taft C, Bjorkelund C, Frid K, Larsson ME (2015). Person-centred care after acute coronary syndrome, from hospital to primary care - a randomised controlled trial. Int J Cardiol.

[14] Lotzke H, Brisby H, Gutke A, Hagg O, Jakobsson M, Smeets R (2019). A person-centered Prehabilitation program based on cognitive-behavioral physical therapy for patients scheduled for lumbar fusion surgery: a randomized controlled trial. Phys Ther.

[15] Jackson T, Wang Y, Wang Y, Fan H (2014). Self-efficacy and chronic pain outcomes: a meta-analytic review. J Pain.

[16] Bernhardsson S, Larsson MEH, Johansson K, Öberg B (2017). “In the physio we trust”: a qualitative study on patients’ preferences for physiotherapy. Physiother Theory Pract.

[17] Larsson ME, Kreuter M, Nordholm L (2010). Is patient responsibility for managing musculoskeletal disorders related to self-reported better outcome of physiotherapy treatment?. Physiother Theory Pract.

[18] Larsson ME, Nordholm LA, Ohrn I (2009). Patients’ views on responsibility for the management of musculoskeletal disorders--a qualitative study. BMC Musculoskelet Disord.

[19] Andersson J, Ahgren B, Axelsson SB, Eriksson A, Axelsson R (2011). Organizational approaches to collaboration in vocational rehabilitation—an international literature review. Int J Integr Care.

[20] Nilsing E, Söderberg E, Öberg B (2012). Sickness certificates in Sweden: did the new guidelines improve their quality?. BMC Public Health.

[21] Cullen KL, Irvin E, Collie A, Clay F, Gensby U, Jennings PA (2018). Effectiveness of workplace interventions in return-to-work for musculoskeletal, pain-related and mental health conditions: an update of the evidence and messages for practitioners. J Occup Rehabil.

[22] Brunner E, De Herdt A, Minguet P, Baldew SS, Probst M (2013). Can cognitive behavioural therapy based strategies be integrated into physiotherapy for the prevention of chronic low back pain? A systematic review. Disabil Rehabil.

[23] Wilson S, Cramp F (2018). Combining a psychological intervention with physiotherapy: a systematic review to determine the effect on physical function and quality of life for adults with chronic pain. Phys Ther Rev.

[24] Hoffman BM, Papas RK, Chatkoff DK, Kerns RD (2007). Meta-analysis of psychological interventions for chronic low back pain. Health Psychol.

[25] Williams AC, Eccleston C, Morley S (2012). Psychological therapies for the management of chronic pain (excluding headache) in adults. Cochrane Database Syst Rev.

[26] Linton SJ, Nicholas M, MacDonald S (2011). Development of a short form of the Orebro musculoskeletal pain screening questionnaire. Spine (Phila Pa 1976).

[27] Ekman I, Swedberg K, Taft C, Lindseth A, Norberg A, Brink E (2011). Person-centered care--ready for prime time. Eur J Cardiovasc Nurs.

[28] Karlson B, Jonsson P, Palsson B, Abjornsson G, Malmberg B, Larsson B (2010). Return to work after a workplace-oriented intervention for patients on sick-leave for burnout--a prospective controlled study. BMC Public Health.

[29] Sennehed CP, Holmberg S, Axen I, Stigmar K, Forsbrand M, Petersson IF (2018). Early workplace dialogue in physiotherapy practice improved work ability at 1-year follow-up-WorkUp, a randomised controlled trial in primary care. Pain..

[30] Hägglund P, Johansson P (2006). Sjukskrivningarnas anatomi – en ESO-rapport om drivkrafterna i sjukförsäkringssystemet.

[31] Swedish, Social, Insurance, Agency (2016). Förslag på utfallsmått för att mäta återgång i arbete efter sjukskrivning [in Swedish]. Social Insurance Report.

[32] Ilmarinen J (2007). The work ability index (WAI). Occup Med.

[33] Ahlstrom L, Grimby-Ekman A, Hagberg M, Dellve L (2010). The work ability index and single-item question: associations with sick leave, symptoms, and health--a prospective study of women on long-term sick leave. Scand J Work Environ Health.

[34] Farrar JT, Young JP, LaMoreaux L, Werth JL, Poole RM (2001). Clinical importance of changes in chronic pain intensity measured on an 11-point numerical pain rating scale. Pain..

[35] Hjermstad MJ, Fayers PM, Haugen DF, Caraceni A, Hanks GW, Loge JH (2011). Studies comparing numerical rating scales, verbal rating scales, and visual analogue scales for assessment of pain intensity in adults: a systematic literature review. J Pain Symptom Manag.

[36] Nicholas MK (1989). Self-efficacy and chronic pain, in paper presented at the annual conference of the British Psychological Society, St. Andrews, Scotland.

[37] Nicholas MK, McGuire BE, Asghari A (2015). A 2-item short form of the pain self-efficacy questionnaire: development and psychometric evaluation of PSEQ-2. J Pain.

[38] Beaton DE, Bombardier C, Guillemin F, Ferraz MB (2000). Guidelines for the process of cross-cultural adaptation of self-report measures. Spine (Phila Pa 1976).

[39] Schwarzer R, Jerusalem M, Weinman J, Wright S, Johnston M (1995). Self-efficacy measurement and generalized self-efficacy scale. Measure in health psychology: a user’s portfolio. Casual and control beliefs (pp. 33-39).

[40] Love J, Moore CD, Hensing G (2012). Validation of the Swedish translation of the general self-efficacy scale. Qual Life Res.

[41] Brooks R (1996). EuroQol: the current state of play. Health Policy.

[42] Soer R, Reneman MF, Speijer BL, Coppes MH, Vroomen PC (2012). Clinimetric properties of the EuroQol-5D in patients with chronic low back pain. Spine..

[43] Linton SJ, Boersma K (2003). Early identification of patients at risk of developing a persistent back problem: the predictive validity of the Orebro musculoskeletal pain questionnaire. Clin J Pain.

[44] Zigmond AS, Snaith RP (1983). The hospital anxiety and depression scale. Acta Psychiatr Scand.

[45] Lisspers J, Nygren A, Soderman E (1997). Hospital anxiety and depression scale (HAD): some psychometric data for a Swedish sample. Acta Psychiatr Scand.

[46] Bjelland I, Dahl AA, Haug TT, Neckelmann D (2002). The validity of the hospital anxiety and depression scale. An updated literature review. J Psychosom Res.

[47] Hensing G (2004). Swedish council on technology assessment in health care (SBU). Chapter 4. Methodological aspects in sickness-absence research. Scand J Public Health Suppl.

[48] Holmgren K, Hensing G, Bultmann U, Hadzibajramovic E, Larsson MEH (2019). Does early identification of work-related stress, combined with feedback at GP-consultation, prevent sick leave in the following 12 months? A randomized controlled trial in primary health care. BMC Public Health.

[49] Oakley A, Strange V, Bonell C, Allen E, Stephenson J, Team RS (2006). Process evaluation in randomised controlled trials of complex interventions. BMJ..

[50] Busch H, Bjork Bramberg E, Hagberg J, Bodin L, Jensen I (2018). The effects of multimodal rehabilitation on pain-related sickness absence - an observational study. Disabil Rehabil.

[51] Hellman T, Bonnevier H, Jensen I, Hagberg J, Busch H, Björk Brämberg E (2014). En processutvärdering av multimodala team inom för rehabiliteringsgarantin. Slutrapport Stockholm.

[52] Severinsson Y, Nordeman L, Holmgren K, Dottori M, Larsson ME (2014). Delrapport 1. Verksamhetsuppföljning av multimodal rehabilitering i primärvård, Västra Götalandsregionen, år 2013.

[53] Saha S, Grahn B, Gerdtham UG, Stigmar K, Holmberg S, Jarl J (2019). Structured physiotherapy including a work place intervention for patients with neck and/or back pain in primary care: an economic evaluation. Eur J Health Econ.

[54] Desiron HA, de Rijk A, Van Hoof E, Donceel P (2011). Occupational therapy and return to work: a systematic literature review. BMC Public Health.

[55] Nazarov S, Manuwald U, Leonardi M, Silvaggi F, Foucaud J, Lamore K (2019). Chronic diseases and employment: which interventions support the maintenance of work and return to work among workers with chronic illnesses? A systematic review. Int J Environ Res Public Health.

